# Immunologic Effects of a Novel Bovine Lactoferrin-Derived Peptide on the Gut and Clinical Perspectives

**DOI:** 10.3390/vetsci11110545

**Published:** 2024-11-06

**Authors:** Haiyue Cui, Huan Yang, Xiaoxi Qi, Yang Zhao, Tianle Huang, Liguang Miao

**Affiliations:** Institute of Special Animal and Plant Sciences, Chinese Academy of Agricultural Sciences, Changchun 130112, China; 82101222393@caas.cn (H.C.);

**Keywords:** bovine lactoferrin-derived peptide, macrophages, immune-enhancing effects, intestinal mucosal immunity

## Abstract

Bovine lactoferrin is a natural iron-binding glycoprotein known for its antimicrobial, antiviral, antitumor, anti-inflammatory, and immunomodulatory properties. In this study, we artificially recombined a fragment of bovine lactoferrin with immunomodulatory and antimicrobial properties to create a novel peptide named LF-MQL. The primary objective was to investigate the effects of LF-MQL on the intestinal tract and immune cells in animals. First, we assessed the in vitro activation effects of LF-MQL on mouse peritoneal macrophages. The results indicated that LF-MQL enhanced the macrophage phagocytic activity and increased IL-1β mRNA expression without significantly affecting IL-6 mRNA levels. Next, we examined the effects of LF-MQL on mucosal immunity by administering LF-MQL orally at doses of 300 mg/kg, 30 mg/kg, and 3 mg/kg to mice. The results demonstrated that different doses of LF-MQL modulated IL-6 and IL-10 mRNA levels in the small intestine. Low doses enhanced the intestinal immune response, while higher doses reduced the inflammatory response. In conclusion, LF-MQL exerts immunomodulatory effects rather than simply boosting immune activity in animal models.

## Simple Summary

A novel peptide derived from bovine lactoferrin has been developed by artificially combining functional fragments with antimicrobial and immunomodulatory activities. This study aimed to investigate the activating effect of this peptide on mouse immune cells and its immune-enhancing impact on the intestine. The results showed that the peptide enhanced macrophage phagocytosis and increased IL-1β mRNA production. Additionally, it regulated the expression of IL-6 and IL-10 mRNA in the intestinal tract in a dose-dependent manner, contributing to the modulation of intestinal immunity. We hope that this study will provide a new reference program for the treatment of animal diseases.

## 1. Introduction

Lactoferrin (Lf) was first isolated from milk by Sorensen et al. (in 1939) [1] and successfully purified from bovine milk by Groves et al. (in 1960) [2]. Early research primarily focused on its iron-binding properties, particularly its role in iron transport and metabolism within the body. The discovery of Lf in the secondary granules of neutrophils by Masson et al. [3] (in 1969) expanded the research scope to include its systemic functions, especially in inflammation and immunity. In 1993, Yamauchi et al. [4] reported that lactoferricin (Lfcin), a derivative peptide of Lf, exhibited stronger antibacterial and antifungal activities than the intact Lf. Further research by Vorland et al. (in 1998) [5] demonstrated that bovine lactoferricin (bLfcin) was the most effective among various species, showing a wide range of biological activities, including antibacterial, antiviral, antitumor, anti-inflammatory, and immunomodulatory effects [6,7,8,9,10,11].

Macrophages (Mф) are key antigen-presenting cells known for their high phagocytic activity [12]. They can be classified into the following two types: M1 macrophages, which possess strong endocytic and antimicrobial capabilities and elicit potent Th1 immune responses, and M2 macrophages, which support Th2-related effector functions and play a role in resolving inflammation [13]. Lactoferrin interacts directly with antigen-presenting cells (APCs) due to the presence of lactoferrin receptors (LfRs) on most immune cells, with the first mammalian LfR identified by Van Snick et al. in 1976 [14].

Intestinal mucosal immunity is the body’s first line of defense against infections, and is crucial for maintaining intestinal integrity and preventing microbial invasion [15,16]. Lactoferrin’s role in mucosal immunity is supported by the presence of intestinal receptors, first proposed by Cox et al. in 1979 [17]. Studies have demonstrated that orally administered bovine lactoferrin is stable and exerts immunomodulatory effects in inflammatory bowel disease, colitis, and intestinal microbiota regulation. Furthermore, recent research highlights its inhibitory role in colorectal cancer due to its ability to suppress excessive cell proliferation. Despite this, research on lactoferrin’s effects on normal immune cells and the intestinal immune system remains limited.

Based on extensive research on bLfcin, our team developed a novel peptide with potent immunomodulatory and antibacterial properties, named LF-MQL, with a molecular weight of 10KD [18]. We have previously investigated the antimicrobial capacity of LF-MQL, focusing on the effects of LF-MQL on chicks infected with Salmonella pullorum and mice infected with Salmonella typhimurium. It was found that LF-MQL was able to reduce the mortality rate of Salmonella-infected chicks and mice without causing toxic side effects in mice [19,20]. In addition, we investigated the in vitro bacteriostatic effect of LF-MQL by incubating it with Staphylococcus aureus and Pseudomonas aeruginosa. We found that the mechanism of its bacteriostatic effect on bacteria was mainly through the disruption of bacterial cell membranes [21]. This study aims to further explore the intestinal immune mechanisms of LF-MQL by examining its effects on macrophages and intestinal immune factors.

## 2. Materials and Methods

### 2.1. Materials and Reagents

LF-MQL and GS_0612_ were provided by the Veterinary Biologics Creation Platform, Institute of Special Animal and Plant Sciences, Chinese Academy of Agricultural Sciences, Changchun, China. The MGY medium and cDNA synthesis kit were purchased from Huayueyang (Beijing, China). DMEM was purchased from Sigma (Shanghai, China). Bovine calf serum, PBS, 100 U/mL penicillin, and 100 U/mL streptomycin were purchased from Solarbio (Beijing, China). DMSO and MTT were purchased from Beyotime (Shanghai). Trizol was purchased from Invitrogen (Beijing, China). A ChamQ Universal SYBR qPCR Master Mix was purchased from Vazyme (Nanjing, China). The microplate reader was purchased from BioTek (Synergy HT, Winooski, VT, USA). Real-time fluorescence quantitative PCR was purchased from Thermo Fisher (Quant Studio3, Waltham, MA, USA).

### 2.2. Preparation of LF-MQL

Saccharomyces cerevisiae strain GS_0612_ was used as the carrier and inoculated in an MGY medium for the fermentation culture. After 30 h of methanol-induced expression, the supernatant was centrifuged at 7000 rpm for 10 min to obtain the target solution. The solution was filtered using a 0.2 µm pore size membrane and then obtained by ion exchange chromatography. The target solution was tested for purity by liquid chromatography. The target activity was qualitatively tested by the tube and dish method using *Escherichia coli* as the indicator bacteria.

### 2.3. Isolation of Peritoneal Macrophages

Peritoneal macrophages (PMф) were obtained by following the previously reported methods. Briefly, peritoneal macrophages were harvested from SPF mice (6–8 weeks old) two days after an intraperitoneal injection of 1 mL of 6% starch broth medium. After sacrificing the mice, 5 mL of sterile PBS was injected into the abdominal cavity. The abdomen was then gently massaged for 3 min, and the peritoneal fluid was subsequently collected. The fluid was centrifuged at 1500 rpm for 10 min, and the resulting cell pellets were resuspended in DMEM supplemented with 10% (*v*/*v*) bovine calf serum, 100 U/mL penicillin, and 100 U/mL streptomycin. The cells were seeded onto plates at a density of 5 × 10^5^ cells/mL and allowed to adhere for 3 h in a humidified incubator at 37 °C with 5% CO_2_. After 4 h of incubation, the non-adherent cells were removed by washing twice with PBS, and fresh medium was added [22].

### 2.4. Cytotoxicity of LF-MQL on Mф

Peritoneal macrophages were collected and purified as described above. The macrophages were plated into a 96-well plate at a density of 2 × 10^5^ cells per well and cultured for 24 h at 37 °C in a humidified atmosphere with 5% CO_2_. LF-MQL was introduced to the cells at concentrations ranging from 0 to 700 μg/mL cells cultured in this DMEM medium without LF-MQL were used as the control). After 48 h of incubation, 30 μL of MTT solution (5 mg/mL) was added to each well 4 h before the end of the incubation. Next, 100 μL of DMSO was added to each well to dissolve the formazan crystals. The plates were shaken for 10 min to ensure the complete dissolution of the crystals. The absorbance was measured using a microplate reader at 570 nm [23].

### 2.5. Pinocytic Activity of Mф

The effects of LF-MQL on the pinocytic activity of PMф cells were determined using the neutral red uptake method, as described in the previous literature [22,23]. Briefly, PMф cells (1 × 10^5^ cells/mL) were suspended in DMEM and placed in a 96-well plate. The cells were treated with LF-MQL (15.625 μg/mL) and bLf (15.625 μg/mL), and an equal amount of DMEM was added to the control group. After culturing for 12 h, the supernatant was replaced with 100 μL of 0.1% neutral red solution. After 4 h of incubation, the cells were washed three times with 0.1 M PBS to remove excess neutral red. Finally, 100 μL of cell lysis solution (ethanol/acetic acid, 1:1, *v*/*v*) was added to each well. The absorbance of each well was measured at 540 nm using a microplate reader.

### 2.6. Measurement of IL-6 and IL-1β mRNA Expression by Mф

After the extraction of mouse peritoneal macrophages, they were inoculated into 6-well plates and cultured in an incubator at 37 °C with 5% CO_2_ for 4 h. A volume of 1 mL of 15.625 μg/mL LF-MQL was added and cultured for 6 h. The total RNA of the peritoneal macrophages was extracted using Trizol, and the cDNA was synthesized using a reverse transcription kit, according to the primer sequences shown in Table 1 (all the primers were designed through the NCBI website). The primers were synthesized according to the primer sequences shown in Table 1 by GENEWIZ (Tianjin, China), and they were mixed well with 1μl of cDNA per well using a ChamQ Universal SYBR qPCR Master Mix, according to the manufacturer’s instructions. Then, a real-time fluorescence quantitative PCR analysis was performed according to the reaction conditions, as shown in Table 2, and the ΔΔCt method was used to calculate the fold change of the gene expression.

### 2.7. Animals and Experimental Design

Female ICR mice (6–8 weeks old), weighing 30.0 ± 2.0 g, were purchased from Liaoning Changsheng biotechnology (China). All mice were kept at a constant temperature of 25 °C under a 12 h light–dark cycle and provided with water and standard chow.

After adaption for a week, the mice were randomly divided into six groups of ten mice in each group. Referring to both the previous literature [20,24] and the actual experimental requirements, the mice were grouped and treatments administered as follows: (1) 300 mg/kg LF-MQL treatment group (300 mg/kg); (2) 30 mg/kg LF-MQL treatment group (30 mg/kg); (3) 3 mg/kg LF-MQL treatment group (3 mg/kg); and (4) the untreated group (control). The mice were treated with oral LF-MQL according to these groupings. LF-MQL was administered as an oral deposition at these concentrations for 7 and 14 days. Three hours after the last administration, the mice were anesthetized, decapitated, and sacrificed, and the small intestine was removed and washed three times with PBS for further experimentation.

### 2.8. Determination of SIgA, NF-кB, IL-6 and IL-10 mRNA Expression in the Small Intestine

The mouse intestines were first treated according to the method described in Section 2.5, where the intestines were sufficiently ground. The total RNA was then extracted from the intestines using Trizol reagent. Following extraction, the cDNA was synthesized using a reverse transcription kit. The synthesized cDNA was subsequently analyzed by real-time fluorescence quantitative PCR (RT-PCR) using a ChamQ Universal SYBR qPCR Master Mix (Vazyme) according to the manufacturer’s instructions. The real-time fluorescence quantitative PCR was performed on a QuantStudio 3 system, and the fold change in gene expression was calculated using the ΔΔCt method.

### 2.9. Statistical Analysis

The results are expressed as the mean ± standard deviation (S.D.) values. Statistical analysis was performed by a one-way and two-way analysis of variance (ANOVA) using GraphPad Prism 9, followed by Tukey’s post-hoc test to determine pairwise differences between groups. *p* values < 0.05, 0.01, 0.001, or 0.0001 were considered to indicate statistical significance.

## 3. Results

### 3.1. Preparation and Characterization of LF-MQL

**Figure 1:**
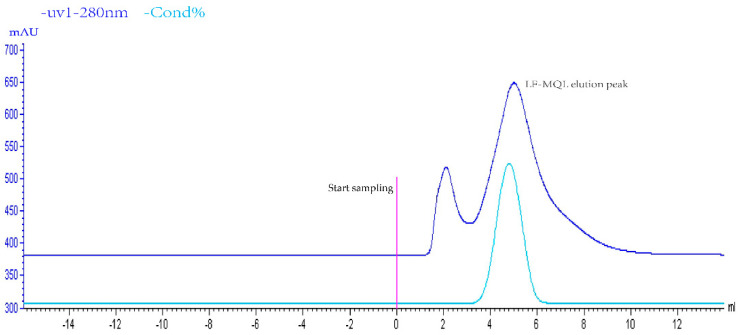
Protein Purifier Column Chromatography.

**Figure 2:**
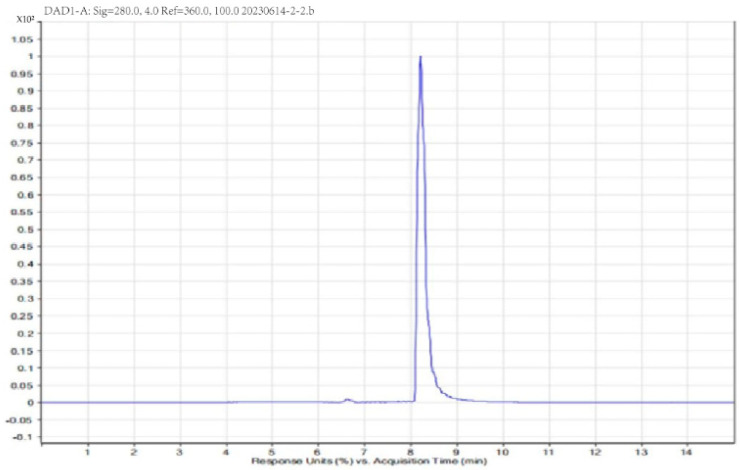
High performance liquid chromatography (HPLC).

**Figure 3:**
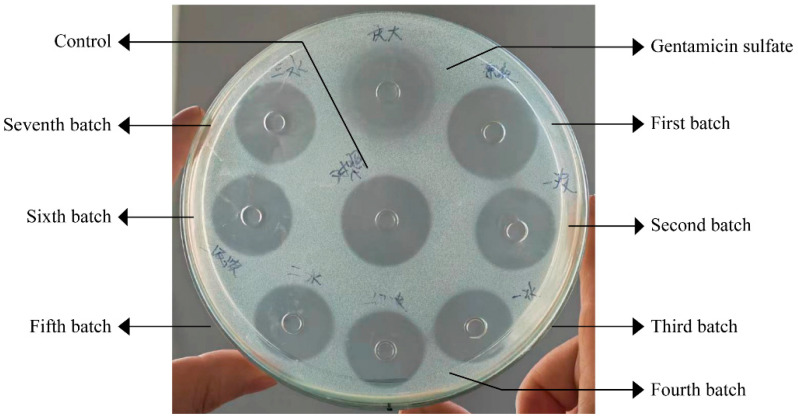
Cap-plate method for the qualitative detection of the inhibitory effect of the target solution on *Escherichia coli*.

The target liquid was obtained by ion exchange chromatography (Figure 1); a single peak was detected by liquid chromatography (Figure 2), indicating that the purity of the collected target liquid met the experimental requirements and that there was no contamination by impurities including endotoxin and other substances. The target had obvious antimicrobial activity, as detected by the tube-dish method (Figure 3), indicating that the sample preparation met the requirements.

### 3.2. Evaluation of LF-MQL Cytotoxicity

**Figure 4:**
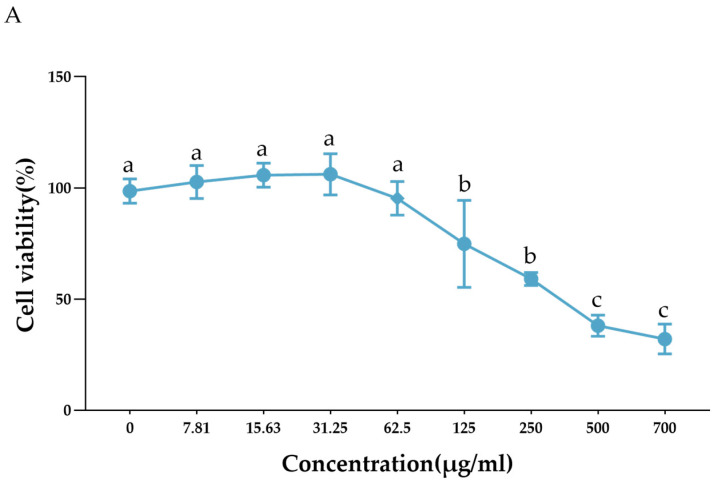

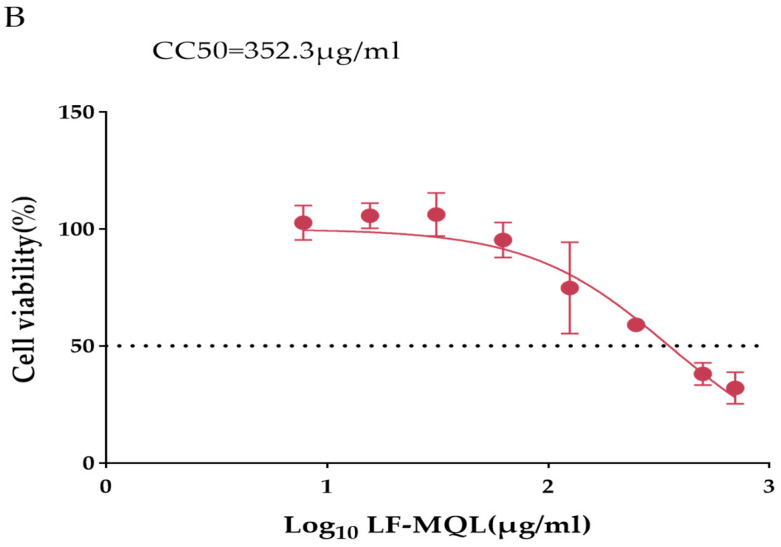
The viability of murine peritoneal macrophages after 24 h of exposure to LF-MQL (**A**) and cytotoxic concentration 50% (CC50) (**B**). Data are presented as mean values ± SD (*n* = 6). ^a–c^ Bars in the figure without the same superscripts differ significantly (*p* < 0.05). Same letters indicate non-significant differences, and different letters indicate significant differences.

The cytotoxicity of LF-MQL toward peritoneal macrophages was assessed using the MTT assay, with macrophages exposed to LF-MQL at concentrations ranging from 0 to 700 μg/mL. As shown in Figure 4A, LF-MQL significantly reduced the cell viability at concentrations of 125 μg/mL and higher, with cell viability dropping to 39.32% at 500 μg/mL. The cytotoxic concentration 50% (CC50) value of 352.3 μg/mL was calculated using GraphPad Prism 9 (Figure 4B). Based on these results, the concentration range of 7.81–62.5 μg/mL was selected for subsequent in vitro macrophage assays due to its moderate toxicity. These results indicated the optimal concentration range for studying LF-MQL’s effects on mouse peritoneal macrophages in vitro.

### 3.3. Pinocytic Activity of Peritoneal Macrophages

**Figure 5:**
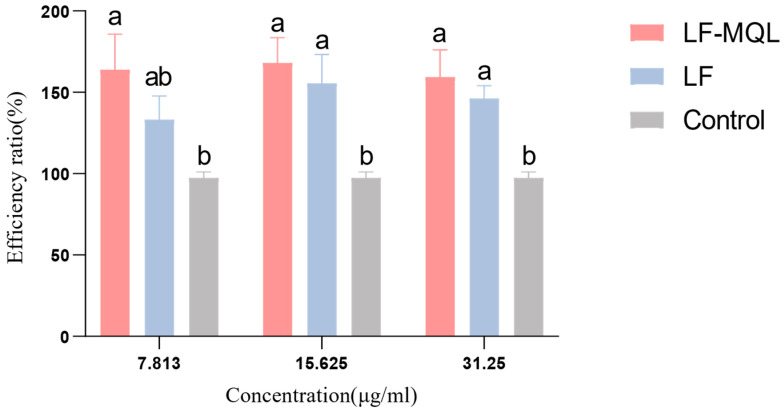
Effects of three concentrations of LF-MQL on the phagocytosis of normal Mф cells. Data are presented as mean values ± SD (*n* = 6). Same letters indicate non-significant differences, and different letters indicate significant differences.

Pinocytic activity is a key indicator of macrophage function. The effect of LF-MQL on the phagocytic function of peritoneal macrophages is shown in Figure 5. Compared to the normal control group, all LF-MQL-treated groups exhibited significantly enhanced phagocytic activity. Notably, the low-concentration LF-MQL group (7.813 μg/mL) showed the most marked increase in the phagocytic rate. Although the medium- (15.625 μg/mL) and high-concentration (31.25 μg/mL) groups also significantly enhanced the phagocytic activity, their effects were less pronounced than in the low-concentration group.

### 3.4. mRNA Expression of IL-6 and IL-1β, by Macrophages After LF-MQL Treatment

**Figure 6:**
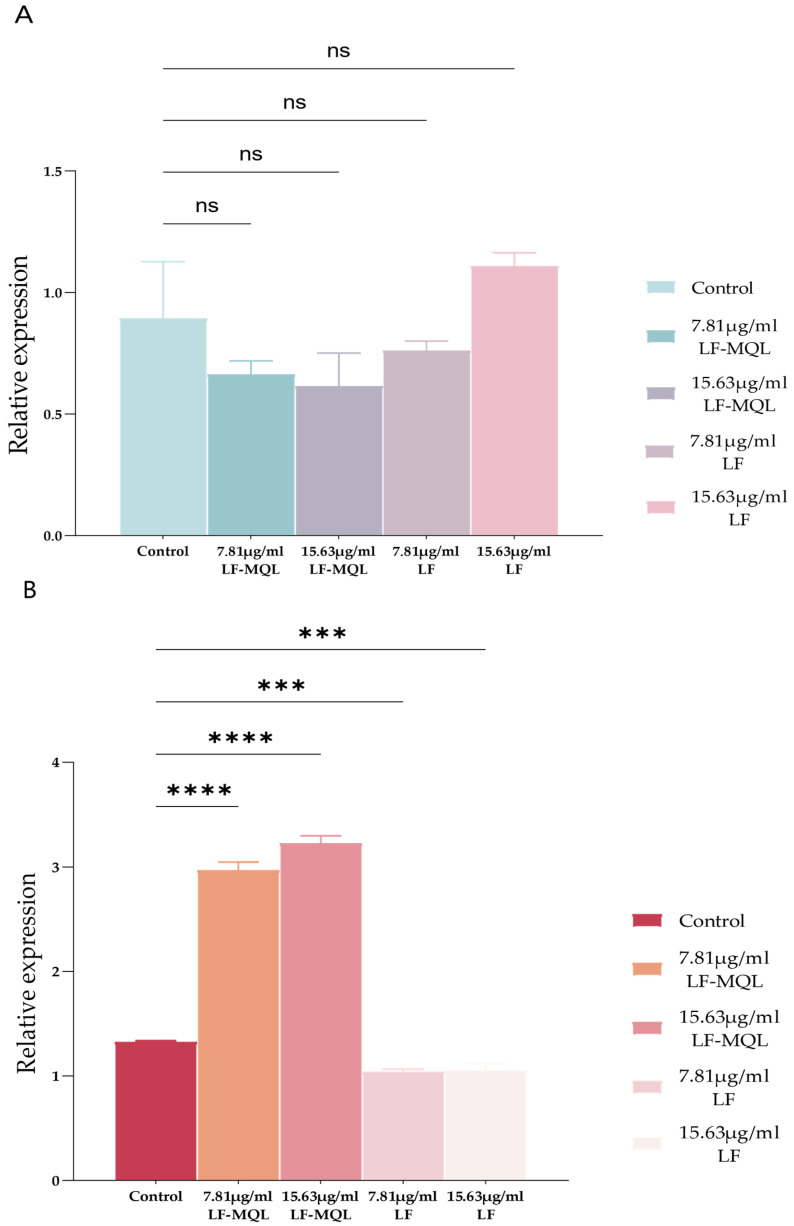
Changes in IL-6 (**A**) and IL-1β (**B**) mRNA expression in each group. Data are expressed as mean values ± SD (*n* = 6). “ns”: no significant difference, *** *p* < 0.001, **** *p* < 0.0001.

To investigate the effect of LF-MQL stimulation on macrophage cytokine production, the mRNA levels of IL-6 and IL-1β were measured. As shown in Figure 6, compared with the untreated control group, none of the abdominal macrophages showed significant changes in IL-6 mRNA expression (Figure 6A) under an in vitro stimulation with LF-MQL (7.81 and 15.63 μg/mL), but all of them showed a highly significant increase in IL-1β (Figure 6B) mRNA expression. The LF group (7.81 and 15.63 μg/mL) showed no significant change in IL-6 mRNA expression, but the LF group’s (7.81 and 15.63 μg/mL) IL-1β mRNA expression was highly significantly decreased. However, there was no significant difference in IL-6 mRNA levels between the LF-MQL-treated, blank, and Lf groups. These findings suggested that LF-MQL primarily stimulates IL-1β mRNA levels and activates macrophages, while its effect on IL-6 mRNA expression is minimal.

### 3.5. Effects of LF-MQL on SIgA and NF-кB mRNA Expression

**Figure 7:**
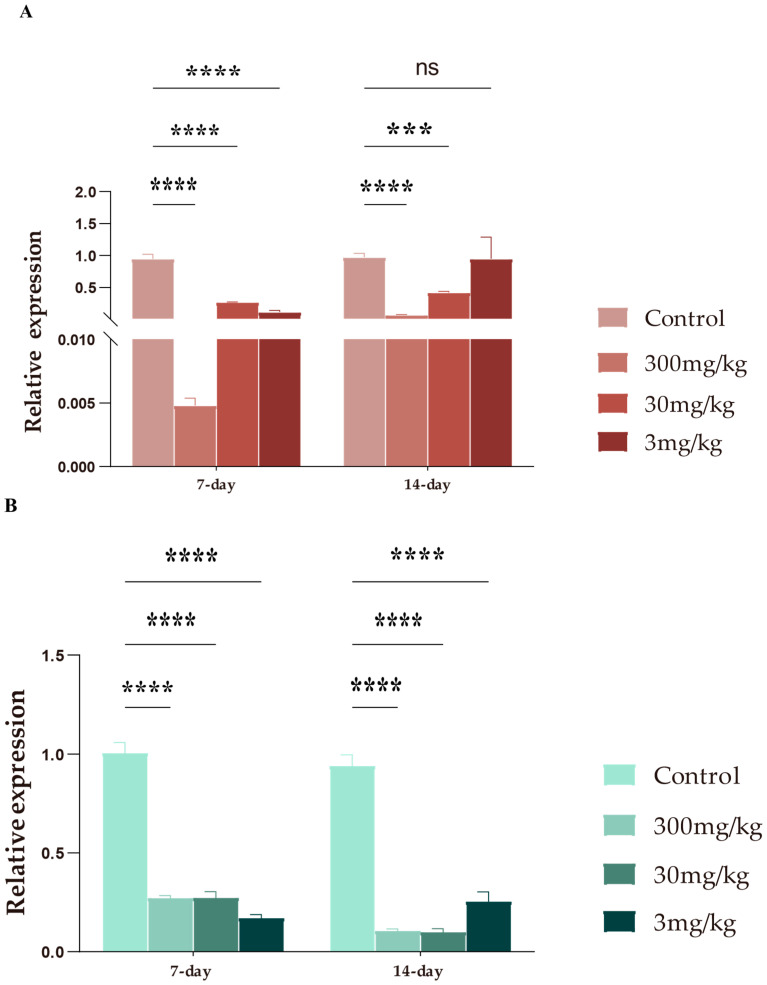
Effects of control, 300 mg/kg, 30 mg/kg, and 3 mg/kg on SIgA (**A**) and NF-кB (**B**) mRNA levels in the small intestine of LF-MQL-treated mice. Data are expressed as mean values ± SD (*n* = 6). “ns”: no significant difference, *** *p* < 0.001, **** *p* < 0.0001.

Secretory IgA (SIgA) plays a crucial role in mucosal immunity by preventing pathogen translocation across the mucosal barrier. As shown in Figure 7, LF-MQL treatments at all tested doses (300 mg/kg, 30 mg/kg, and 3 mg/kg) significantly decreased intestinal SIgA (Figure 7A) mRNA levels after 7 days, with a similar trend observed after 14 days at 300 mg/kg and 30 mg/kg. However, the 3 mg/kg group showed no significant change in SIgA mRNA levels after 14 days. Additionally, LF-MQL significantly reduced NF-кB (Figure 7B) mRNA levels in the intestines of mice at all doses and time points tested.

### 3.6. mRNA Expression of IL-6 and IL-10 by Intestinal Tissue After LF-MQL Treatment

**Figure 8:**
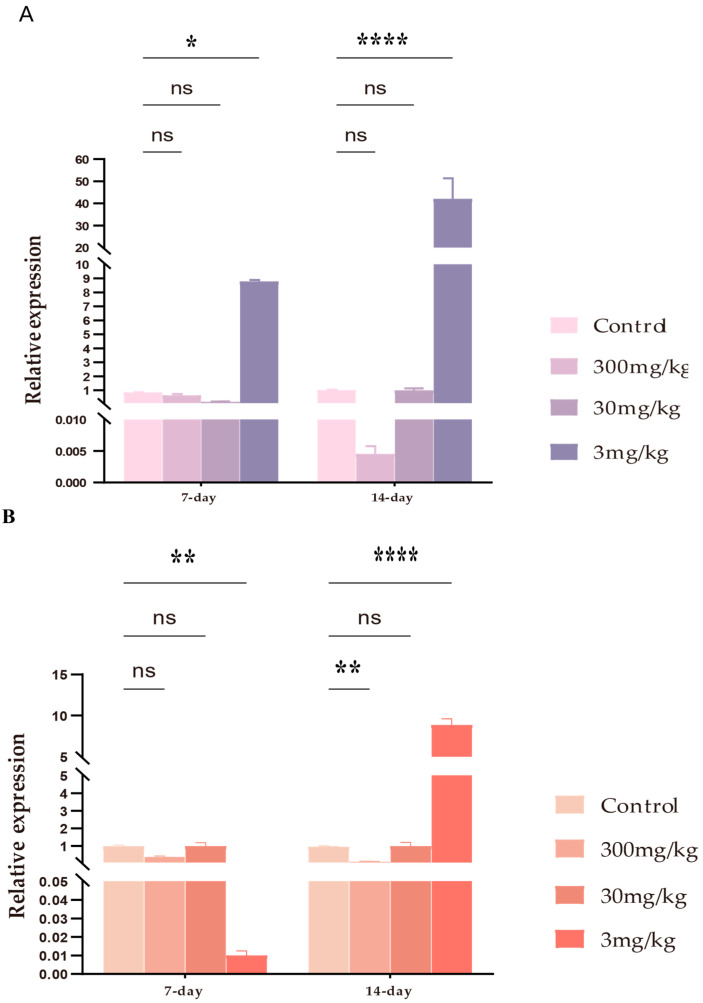
Effects of control, 300 mg/kg, 30 mg/kg, and 3 mg/kg on IL-6 (**A**) and IL-10 (**B**) mRNA levels in the small intestine of LF-MQL-treated mice. Data are expressed as mean values ± SD (*n* = 6). “ns”: no significant difference, * *p* < 0.05, ** *p* < 0.01, **** *p* < 0.0001.

Mucosal immune cytokines, including IL-6 and IL-10, play essential roles in intestinal immunity. Compared with the normal control group for IL-6 mRNA levels (Figure 8A), no significant changes were observed in the other groups (300 mg/kg, 30 mg/kg) after 7 days of treatment with different concentrations of LF-MQL, except for a significant increase in the 3 mg/kg LF-MQL treatment, and a significant increase after 14 days in the 3 mg/kg group. No significant changes were noted in the IL-6 mRNA levels in the other dose groups after 14 days. For IL-10 mRNA levels (Figure 8B), there was no significant change in mRNA levels after 7 days at 300 mg/kg and 30 mg/kg, but a significant decrease was observed at 3 mg/kg. After 14 days, the IL-10 mRNA levels significantly decreased at 300 mg/kg, while the 3 mg/kg group showed a very significant increase. No significant change was observed at 30 mg/kg.

## 4. Discussion

Lactoferrin (Lf) belongs to the transferrin family and is a single polypeptide chain glycoprotein with a molecular weight of about 80 kDa [25]. It is mainly found in body fluids such as mammalian milk, saliva, and semen, as well as on mucosal surfaces and in polymorphonuclear leukocyte granules [24]. Among these, human and cow’s milk are the most abundant sources of Lf. As a key molecule of the innate non-specific immune system, Lf can modulate immune responses according to the host’s immune status, thereby leading to varying physiological effects based on its concentration, purity, and source.

Our experiments demonstrated that LF-MQL significantly enhanced macrophage pinocytosis, which aligns with previous studies on bovine lactoferrin peptides [26]. The enhancement of macrophage phagocytosis helps the body to fight against microbial infections and cancers. In vitro stimulation of macrophages by LF-MQL (31.25 μg/mL, 15.625 μg/mL, and 7.813 μg/mL) significantly enhanced its pinocytosis activity, thus enhancing the body’s ability to fight against foreign invasions and improving the body’s immunity. However, while LF-MQL significantly impacted IL-1β mRNA expression by macrophages, its effect on IL-6 mRNA expression was not statistically significant, indicating that LF-MQL primarily stimulates IL-1β mRNA expression and macrophage activation rather than IL-6 mRNA expression. Macrophages (Mф) are highly phagocytic cells that play a crucial role in natural immunity by engulfing pathogenic microorganisms, and they are also involved in humoral and cellular immunity. Lf can interact directly with Mф via nucleolin, a multifunctional shuttle protein on the surface of Mф [22,27]. Some immunomodulatory effects of Lf depend on its ability to form complexes with LPS. Direct exposure of Mф to bLf leads to the production of cytokines such as IL-6, IL-8, IL-12, IFN-α, and IFN-γ, although its effect on activated Mф is less pronounced [28,29,30]. Additionally, Lf can influence the classical and alternative complement pathways, with the Lfc structural domain of hLf or bLfc inhibiting the classical pathway but not the alternative pathway. Thus, the mechanism by which LF-MQL enhances the phagocytosis of mice PMф requires further investigation.

In our study, we observed that different concentrations of LF-MQL either increased or decreased the mRNA levels of IL-6 and IL-10 in the small intestine, with 3 mg/kg of LF-MQL significantly enhancing both cytokines after 14 days of treatment. These results suggested a complex regulation of the immune response in the gut, where the balance between pro-inflammatory and anti-inflammatory factors is critical for maintaining intestinal homeostasis. IL-6 plays a dual role in inflammation and anti-inflammation, acting as a mediator between immune cells and gut microbes [31,32]. IL-10, an important anti-inflammatory cytokine, inhibits the secretion of many pro-inflammatory cytokines and chemokines and is a major regulator of the inflammatory response in the intestinal tract. IL-10 and IL-6 act mainly through the same signaling pathway, and both cytokines activate the signal transducer and activator of transcription (STAT3) [33,34]. Although pro-inflammatory cytokines contribute to host defense, their overproduction can lead to excessive inflammatory responses, tissue destruction, and septic shock. Therefore, the balance between pro-inflammatory and anti-inflammatory cytokines profoundly influences the development of the inflammatory response and the role of immunomodulation. Interestingly, the increase in IL-10 mRNA levels observed in the intestines of LF-MQL-treated mice also revealed that LF-MQL may play a regulatory or therapeutic role in allergic and hypersensitivity reactions.

It has been shown that spontaneous colitis is exhibited in IL-10^−/−^ mice [35], but colitis does not occur in germ-free IL-10^−/−^ mice [36], suggesting that the presence of IL-10 inhibits the development of colitis caused by harmful intestinal bacteria [37]. A growing body of research suggests a protective role for IL-10 in colitis induced by a variety of factors, particularly in the prevention of inflammatory bowel disease (IBD). Chronic inflammation is a contributing factor to the development of cancer, and studies have shown that people with IBD have a significantly increased chance of developing cancer [38]. It has been found that Lf can inhibit tumor growth through MAPK and AKT signaling pathways, and at the same time MAPK is one of the key factors regulating IL-10 [39]. Increasing evidence suggests that the overdevelopment of inflammatory responses may be one of the factors leading to tumorigenesis. Therefore, anti-inflammatory factors, including IL-10, become important targets for tumor prevention and treatment. IL-10 can enhance the immune response and promote anti-proliferative and pro-apoptotic pathways by activating the STAT1 pathway in tumor-resident cells CD8^+^T [40]. In our study, we found that LF-MQL inhibited the activation of NF-кB but significantly increased the expression of IL-10 mRNA. Therefore, based on the results of this study, we can reasonably deduce that LF-MQL can play a positive role in inhibiting the development of cancer and in the treatment of cancer at a certain dosage. However, based on the limitations of this study, the specific mechanism of the action of LF-MQL in the development and treatment of cancer still needs to be further investigated. 

SIgA, the major antibody isotype in the mucosal immune system, is predominantly present as secretory IgA (SIgA) at mucosal sites, where it is considered a non-inflammatory antibody [41]. B cells acquire the ability to produce SIgA through class-switch recombination, which can occur via T-cell-dependent (TD) or T-cell-independent (TI) pathways. Peyer’s patches (PPs) are a major source of precursors for IgA+ plasma cells (PCs) [42]. It is well known that SIgA secretion is low in germ-free mice, but rapidly returns to normal levels after bacterial colonization. In previous studies involving the oral administration of bovine lactoferrin peptides to animals, intestinal SIgA levels were largely elevated. This research suggests that the increase in SIgA levels observed in the intestines of LF-MQL-treated mice may be associated with alterations in the intestinal microflora. Additionally, SIgA secretion in the intestines of most mammals is also regulated by the NF-кB signaling pathway [43]. NF-кB-inducible kinase (NIK) is indispensable for mediating cellular immunity and the formation of secondary lymphoid tissues (SLTs) [44]. Atypical NIK-induced NF-кB signaling enhances the expression of polymeric immunoglobulin receptors (pIgRs) in the intestinal epithelium by inducing IL-23 expression in dendritic cells (DCs), leading to an increase in microbiota-independent IgA secretion into the intestinal lumen. In our study, intestinal NF-кB levels were significantly reduced in LF-MQL-treated mice, which supports the reliability of our findings regarding SIgA mRNA levels. It is hypothesized that the decrease in SIgA mRNA levels observed in the intestines of LF-MQL-treated mice may be related to the modulation of NF-кB signaling, but the exact mechanism of action needs further investigation. It is also well known that the activation of NF-кB plays a role in the development of cancer in animal organisms [45,46]. NF-кB activates the transcription of concentrated genes that inhibit cell death through the mitochondrial and death receptor pathways, and it does so by inducing the expression of inhibitors of apoptosis (IAPs) and some members of the anti-apoptotic Bcl-2 family of cells, whose overproliferation is a major cause of cancer development [47]. Meanwhile, previous studies have also found that Lf has an anticancer effect [48], and in this study, LF-MQL significantly reduced NF-кB mRNA levels in the intestines of mice. It may have a stronger anticancer effect, but of course the mechanism of action that produces this effect needs further study.

## 5. Conclusions

In summary, our novel bovine lactoferrin-derived peptides demonstrated enhanced immunoreactivity both in vivo and in vitro in cellular and animal experiments. Specifically, LF-MQL treatments at concentrations of 7.813 μg/mL, 15.625 μg/mL, and 32.25 μg/mL significantly promoted phagocytosis activity in normal macrophages. Among these, 15.625 μg/mL of LF-MQL increased the mRNA levels of IL-1β in macrophages, while it did not significantly affect IL-6 mRNA levels. In animal studies, LF-MQL treatments at doses of 300 mg/kg, 30 mg/kg, and 3 mg/kg led to a significant decrease in the mRNA levels of SIgA and NF-кB in the intestines. However, there was no significant change in SIgA levels after 14 days of treatment. The IL-6 mRNA levels in the intestines significantly increased after 7 and 14 days of treatment with 3 mg/kg LF-MQL. Additionally, the IL-10 mRNA levels in the intestines of mice treated with 3 mg/kg LF-MQL significantly decreased after 7 days but reversed after 14 days of treatment. Therefore, LF-MQL at a dose of 3 mg/kg exhibited a significant immune-enhancing effect on mice, while a higher dose of 300 mg/kg LF-MQL may mitigate or have therapeutic effects on hypersensitivity reactions, offering a novel research direction for future studies. Low-dose LF-MQL promotes the activation of the immune system by enhancing macrophage pinocytosis and cytokine release, thereby enhancing the body’s immune response. However, high doses of LF-MQL inhibit these responses, leading to immune tolerance or suppression. This property of LF-MQL suggests that different doses of LF-MQL should be used to achieve different immune effects.

In addition, this pre-use study of a novel bovine lactoferrin-derived peptide has some research limitations at this time. For example, the role of LF-MQL in cancer is still unknown, and we can only infer that it has preventive and therapeutic effects for cancer based on the current study content. The anti-inflammatory effect of LF-MQL also needs to be studied under an inflammation model. At the same time, the detection of both SIgA and cytokines in this study was done by RT-PCR to detect their mRNA levels without detecting their protein levels, so the detection of protein levels of SIgA and cytokines is also the next step in the study. It is these limitations that provide the direction for our next research!

## Figures and Tables

**Table 1:** Mouse primers used in this study.

Name	Primer	Sequence (5′-3′) of Primer	Size (bp)
IL-6	IL-6-F	TCTGGTCTTCTGGAGTACCATAGC	
	IL-6-R	TGTGACTCCAGCTTATCTCTTGGT	142
IL-1β	IL-1β-F	TGCCACCTTTTGACAGTGATGA	
	IL-1-R	GTTGATGTGCTGCTGCGAGA	140
SIgA	SIgA-F	GGCATGTCAGGGACAAGAG	
	SIgA-R	GGAACAGTGGCGCATCATTC	141
NF-кB	NF-кB-F	ACAAAAACTGGGCCACTCTGG	
	NF-кB-R	TCCCGGAGTTCATCTCATAGTTGT	117
IL-10	IL-10-F	AGGCGCTGTCATCGATTTCTCC	
	IL-10-R	GTAGACACCTTGGTCTTGGAGC	96

**Table 2:** RT-PCR reaction conditions.

Stage 1	pre-denaturation	Rep: 1	95 °C	30 s
Stage 2	cyclic reaction	Reps: 40	95 °C	10 s
			60 °C	30 s
Stage 3	melting curve	Rep: 1	95 °C	15 s
			60 °C	60 s
			95 °C	15 s

## Data Availability

The data presented in this study are available in this article.
